# Inhaled liposomal amphotericin-B as a prophylactic treatment for COVID-19-associated pulmonary aspergillosis/aspergillus tracheobronchitis

**DOI:** 10.1186/s13054-021-03728-w

**Published:** 2021-08-19

**Authors:** Sofie Van Ackerbroeck, Lynn Rutsaert, Ella Roelant, Kathleen Dillen, Joost Wauters, Niels Van Regenmortel

**Affiliations:** 1 Department of Intensive Care Medicine, Ziekenhuis Netwerk Antwerpen Campus Stuivenberg, Lange Beeldekensstraat 267, 2060 Antwerp, Belgium; 2 Department of Haematology, Gasthuiszusters Antwerpen, Campus St. Augustinus, Oosterveldlaan 24, 2610 Wilrijk, Belgium; 3 Center for Statistics, StatUa, University of Antwerp, Prinsstraat 13, 2000 Antwerp, Belgium; 4 Clinical Trial Center (CTC), CRC Antwerp, Antwerp University Hospital, University of Antwerp, Drie Eikenstraat 655, 2650 Edegem, Belgium; 5 Department of Pharmacy, Ziekenhuis Netwerk Antwerpen Campus Stuivenberg, Lange Beeldekensstraat 267, 2060 Antwerp, Belgium; 6 Medical Intensive Care Unit, Department of General Internal Medicine, University Hospitals Leuven, Leuven, Belgium; 7 Department of Intensive Care Medicine, Antwerp University Hospital, Drie Eikenstraat 655, 2650 Edegem, Antwerp Belgium

COVID-19-associated pulmonary aspergillosis (CAPA) is a recently described complication of severe Coronavirus Disease of 2019 and is associated with increased morbidity and mortality [1, 2]. Also Aspergillus tracheobronchitis (AT) is common in this disease. We reported already during the first pandemic wave that CAPA/AT was frequently encountered in our 31-bed mixed ICU (ZiekenhuisNetwerk Antwerpen, Belgium) where we often take care of comorbid and immunocompromized patients, regularly of low socioeconomic status [3]. Since we were dealing with a novel disease and in view of the extensively documented health risks of influenza-associated pulmonary aspergillosis, we decided a few months after the beginning of the pandemic to start with a routine off-label prophylactic regimen in mechanically ventilated patients [4]. We chose inhaled liposomal amphotericin-B in view of its successful and safe use in hematological disease and in solid organ transplant patients [5]. We chose it over prophylactic triazoles to minimize the risk of azole resistance and in view of local reimbursement criteria. Specifically, 12.5 mg Ambisome® (Gilead Sciences, Inc.), dissolved in 3 mL of sterile water with the addition of 5 drops of salbutamol, was nebulized on Mondays and Thursdays using our routine Covidien® DAR® nebulizer set in all intubated patients. The solution was administered as close as possible to the endotracheal tube to avoid precipitate in the circuit. After the nebulization, the expiratory filter was checked for potential clogging.

We performed this retrospective observational study to assess the effectiveness of this prophylactic regimen. We aimed to compare the proportion of patients who protracted proven or probable CAPA/AT while receiving the treatment with the proportion of patients with CAPA/AT who only received standard of care. We also assessed the proportion of patients with Aspergillus colonization in endotracheal aspirates in relation to the treatment. The study was approved by our Institutional Review Board (approval nr. 5530) with waiving of patient consent. We included every patient who had been mechanically ventilated for COVID-19 pneumonia and had undergone a diagnostic procedure for CAPA/AT between the start of the first pandemic wave until March 31, 2021. Patients who protracted invasive pulmonary aspergillosis before ICU admission were excluded. The routine implementation of the prophylactic regimen made this mostly a before and after study, although a few patients did not receive the treatment after its implementation and were analyzed in the standard-of-care group.

During the study period, 203 patients were admitted to our ICU for COVID-19 pneumonia of which 78 needed mechanical ventilation. Not every patient was screened for CAPA/AT: especially in the early phase of the pandemic, physicians were reluctant to perform aerosol-generating diagnostic procedures. In other cases, oxygen need was considered too high to allow a safe procedure. Figure 1 shows the derivation of the study cohort of eventually 50 patients. The patient characteristics are reported in Table 1. Eleven patients who received the standard of care developed CAPA/AT, compared to three of the patients who did receive the prophylactic treatment (risk ratio (RR) was 0.15, 95%CI 0.05 to 0.48; Chi^2^ *p* < 0.001). Median time to diagnosis was 11 days in the treated group (IQR 7–19) and 12 days in the untreated group (IQR 8–18) (*p* = 0.74). Also the proportion of Aspergillus colonization in endotracheal aspirates was significantly lower: 44% (*n* = 8) in the standard of care group compared to 13% (*n* = 4) in the treated group (RR 0.28, 95%CI 0.10 to 0.81; Fisher’s exact *p* = 0.017). No treatment-related adverse events were encountered, including bronchospasms that urged to stop the prophylaxis.

**Fig. 1:**
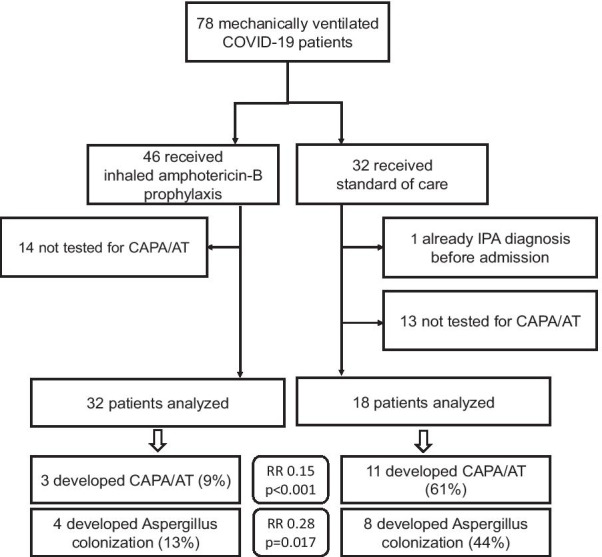
Cohort derivation plot and main findings of this retrospective observational study of the twice weekly prophylactic use of inhaled liposomal amphotericin-B in mechanically ventilated patients with COVID-19 pneumonia. Whenever feasible in view of patient or clinician safety, the patients were screened for CAPA/AT by assessing platelia galactomannan enzyme immunoassay on bronchoscopy-obtained bronchoalveolar lavage fluid. An index of 1 or more in combination with the presence of pulmonary infiltrates on chest radiography or in combination with ulcers, pseudomembranes or airway plaques encountered during bronchoscopy was considered probable CAPA/AT [2]. During the first pandemic wave, before the routine implementation of high-dose thromboprophylaxis, bronchial biopsy was also regularly performed to demonstrate proven IPA [3]. IPA = invasive pulmonary aspergillosis; CAPA/AT = COVID-19-associated pulmonary aspergillosis/Aspergillus tracheobronchitis; RR = risk ratio

**Table 1:** Characteristics of the study cohort

	Overall (n = 50)	Standard of care (n = 18)	Inhaled amphotericin-B (n = 32)	*p*-value
*Baseline characteristics*				
Age	65.5 (10.8)	64.8 (13.0)	65.9 (9.62)	0.75^a^
Male sex	37 (74.0%)	17 (94.4%)	20 (62.5%)	0.02^b^
Immunocompromized state	15 (30.0%)	7 (38.9%)	8 (25.0%)	0.30^c^
Cancer (incl. hematological)	13 (26.0%)	5 (27.8%)	8 (25.0%)	
Immunosuppressive or HIV medication	6 (12.0%)	2 (11.1%)	4 (12.5%)	
Organ transplant	1 (2.0%)	0 (0%)	1 (3.1%)	
Primary immunoglobulin G deficiency	2 (4.0%)	2 (11.1%)	0 (0%)	
*Other comorbidities*				
Diabetes mellitus	26 (52.0%)	8 (44.4%)	18 (56.2%)	0.42^c^
Arterial hypertension	26 (52%)	6 (33.3%)	20 (62.5%)	0.02^c^
Heart failure (NYHA ≥ 2)	2 (4.4%)	1 (5.6%)	1 (3.1%)	1^b^
Chronic kidney disease without dialysis	5 (10%)	2 (11.1%)	3 (9.4%)	1^b^
Chronic dialysis	3 (6%)	1 (5.6%)	2 (6.3%)	1^b^
Modified frailty index [6]	0.16 (0.12)	0.12 (0.11)	0.18 (0.13)	0.14 ^a^
*Admission characteristics*				
PaO_2_/FiO_2_ at admission	111 (76–136)	91 (73–147)	113 (81–136)	0.41^d^
SOFA score at admission	5.5 (4–8)	7 (4–8)	5 (4–8)	0.51 ^d^
SAPS-3 score	55.7 (17.7)	54.0 (12.0)	54.6 (14.1)	0.68 ^a^
Time from hospital admission to intubation (days)	3.5 (1–10)	5.5 (1–16)	3 (1–10)	0.51 ^d^
Time from ICU admission to intubation (days)	2 (0–7)	1.5 (0–8)	2 (0–5)	0.67 ^d^
Vasopressor use	48 (96%)	16 (88.9%)	32 (100%)	0.05 ^c^
Renal replacement therapy (incl. chronic dialysis)	20 (40%)	9 (50.0%)	11 (34.4%)	0.28 ^c^
*COVID-19 treatment*				
Dexamethasone	33 (66.0%)	9 (50.0%)	24 (75.0%)	0.07 ^c^
Tocilizumab	8 (16.0%)	2 (11.1%)	6 (18.8%)	0.69 ^b^

Numbers are presented as means (standard deviation), n (%) or medians (interquartile range) as appropriate. Test statistics: a = independent samples t-test; b = Fisher’s exact test; c = Chi square test; d = Mann–Whitney test

NYHA = New York Heart Association; SAPS = simplified acute physiology score; SOFA = sequential organ assessment score; ICU = intensive care unit; COVID-19 = Coronavirus Disease of 2019

In this observational study, we found that a twice weekly prophylactic regimen of 12.5 mg inhaled liposomal amphotericin-B reduced the incidence of CAPA/AT in mechanically ventilated COVID-19 patients. Confirmation of these findings in a randomized clinical trial is needed.

## Data Availability

The datasets used and/or analyzed during the current study are available from the corresponding author on reasonable request.
